# Alexithymia modulates the experience of the rubber hand illusion

**DOI:** 10.3389/fnhum.2015.00357

**Published:** 2015-06-18

**Authors:** Delphine Grynberg, Olga Pollatos

**Affiliations:** ^1^Psychological Sciences Research Institute, Université Catholique de Louvain and Fonds National de la Recherche ScientifiqueLouvain-la-Neuve, Belgium; ^2^Health Psychology, Institute of Psychology and Education, University of UlmUlm, Germany

**Keywords:** alexithymia, rubber hand illusion, multisensory integration, body ownership, proprioception

## Abstract

Alexithymia is associated with lower awareness of emotional and non-emotional internal bodily signals. However, evidence suggesting that alexithymia modulates body awareness at an external level is scarce. This study aimed to investigate whether alexithymia is associated with disrupted multisensory integration by using the rubber hand illusion task. Fifty healthy individuals completed the Toronto Alexithymia Scale and underwent the rubber hand illusion measure. In this measure, one watches a rubber hand being stroked synchronously or asynchronously with one’s own hand, which is hidden from view. Compared to the asynchronous stimulation, the synchronous stimulation results in the illusion that the rubber hand and the participant’s hand are closer together than they really are and that the rubber hand belongs to them. Results revealed that higher levels of alexithymia are associated with a lower ownership illusion over the rubber hand. In conclusion, our findings demonstrate that high alexithymia scorers integrate two simultaneous sensory and proprioceptive events into a single experience (lower multisensory integration) to a lesser extent than low alexithymia scorers. Higher susceptibility to the illusion in high alexithymia scorers may indicate that alexithymia is associated with an abnormal focus of one’s own body.

## Introduction

An interest in alexithymia originally emerged in the psychosomatic field. In 1973, Sifneos proposed the term “alexithymia” to characterize patients presenting somatization disorders, defined by a high number of somatic complaints and misinterpretations of somatic sensations as signs of physical illness (Sifneos, 1973; Lundh and Simonsson-Sarnecki, 2001). These patients presented lower emotional awareness, more specifically, difficulties in identifying and describing their feelings, with a focus on external events and a poor imagination. Nowadays, there is a consensus in the literature regarding the main components of alexithymia (Taylor et al., 1997): (a) difficulty distinguishing between feelings and bodily sensations accompanying states of emotional arousal, (b) difficulty describing feelings, and (c) externally oriented thinking.

At an empirical level, several studies have suggested that alexithymia is associated with lower awareness of emotional responses. They indeed showed a discrepancy (i.e., over- or underestimation) between the subjective and physiological responses to emotional or stressful stimuli (e.g., Grynberg et al., 2012). In addition to lower emotional awareness, studies in alexithymia have highlighted lower awareness of non-emotional internal bodily experience. High alexithymia scorers are indeed less accurate in reporting the number of perceived heartbeats compared with the actual number of heartbeats that occur (Herbert et al., 2011), which indicates impaired interoceptive awareness. Previous research has also revealed that high scorers report a lower ability to anticipate body signals (i.e., predicting one’s own bodily reactions; Bekker et al., 2008). In addition, high alexithymia scorers report lower familiarity with their body (Carano et al., 2006; De Berardis et al., 2007). Taken together, these studies suggest that alexithymia is characterized by lower awareness of one’s own body.

However, no study has yet examined how alexithymia influences multisensory integration. Specifically, the investigation of the association between alexithymia and the integration of simultaneous processing of tactile, visual and proprioceptive signals is of high importance because the latter is necessary for body awareness. Indeed, it leads to experiencing the body as one’s own and to the feeling that one’s own body parts belong to oneself (e.g., Tsakiris et al., 2011). This integration refers to the concept of *body ownership* and represents a main channel of information available for self-awareness (Tsakiris et al., 2011). As alexithymia has been shown to be associated with impaired body awareness at internal (e.g., interoception) and external (e.g., familiarity) levels, one may hypothesize that alexithymia is also associated with impaired multisensory integration.

Therefore, on the basis of these preliminary data, the present study explored the association between alexithymia and the integration of visual, tactile and proprioceptive signals in order to determine whether alexithymia modulates body ownership. Reporting such integration deficits in alexithymia has strong implications as it determines whether alexithymia is characterized by lower self-awareness in terms of body ownership.

One way to assess multisensory integration is by using the rubber hand illusion (RHI) task (Botvinick and Cohen, 1998). In this measure, one watches a rubber hand being stroked synchronously or asynchronously with one’s own hand, which is hidden from view. At a behavioral level, the synchronous stimulation results in the illusion that the rubber hand and the participant’s hand are closer than they really are. This effect is also known as a proprioceptive drift and is defined as “*the change in the perceived position of the hand between the start and end of the stimulation period, across conditions*” (Tsakiris et al., 2010; p. 346). At a subjective level, participants report the illusion that the rubber hand belongs to them and that their real hand is in the location where the rubber hand actually is. At a physiological level, the illusion is quantified by a drop in the skin temperature of one’s own hand (Moseley et al., 2008). Therefore, at a *bottom-up* level, the illusion arises from the integration between the perception of the stimulation of the non-self body part and the tactile stimulation of the self body part that occur in synchrony (vs. asynchrony). The illusion then induces a stronger sense of body ownership over a fake hand and leads to changes in body image.

In addition to the influence of bottom-up factors (e.g., synchronicity), several studies have demonstrated that body ownership is also influenced by *top-down* factors such as psychiatric disorders (e.g., Autism Spectrum Disorder, ASD; Cascio et al., 2012) or personality traits (e.g., psychosis-proneness; Germine et al., 2013). In the present study, we aimed to investigate whether alexithymia constitutes an additional top-down factor that modulates the RHI. Although this association has not yet been explored, some findings are in line with this hypothesis. For instance, in various disorders characterized by high levels of alexithymia, the RHI is either reduced (ASD; Cascio et al., 2012) or increased (e.g., schizophrenia, eating disorders; Thakkar et al., 2011; Eshkevari et al., 2012).

Based on these studies, three main RHI patterns may be expected in high relative to low alexithymia scorers:

1. If high scorers present higher RHI in synchronous *and* asynchronous conditions, this would suggest that they allocate higher perceptual attention to the rubber hand (Eshkevari et al., 2012). Specifically, this pattern of results would suggest that they present higher sensitivity to visual capture relative to tactile and proprioception information, as already observed in schizophrenia (e.g., Thakkar et al., 2011) and in eating disorders (Eshkevari et al., 2012).
2. If high scorers present higher RHI in the synchronous condition only, this would suggest that they have higher sensitivity to multisensory integration, as found in psychosis proneness (Germine et al., 2013) and in medically unexplained symptoms (Miles et al., 2011).
3. If high scorers present lower RHI in the synchronous condition, this would suggest that they are not influenced by visual information. Lower multisensory integration may thus be due to either a greater reliance on tactile sensory inputs or a greater focus on proprioceptive signals. The absence of RHI during the synchrony condition has been shown in ASD participants during the first 3 min of stimulation (Cascio et al., 2012).

## Method

### Participants

Fifty participants (*M*_age_ = 22.92, *SD*_age_ = 4.04; 43 women) were included in the experiment, which took place at the University of Ulm, Department for Health Psychology. It was conducted in accordance with the Declaration of Helsinki with the approval of the local ethics committee. All participants gave their written informed consent and received class credits for their participation.

### Material

#### Questionnaire

The 20-item Toronto Alexithymia Scale (TAS-20; Bagby et al., 1994) measures three dimensions of the construct: difficulty identifying emotions (DIF; e.g., “I am often confused about what emotion I am feeling”); difficulty describing emotions (DDF; e.g., “It is difficult for me to find the right words for my feelings”); and externally oriented thinking (EOT; e.g., “I prefer talking to people about their daily activities rather than their feelings”).

#### Rubber Hand Illusion

The RHI paradigm is described in detail in the Procedure Section. In the present study, three measures were used for each participant: the subjective reports of the illusion, the proprioceptive shift, and the temperature of the participant’s hand. Subjective scores were evaluated via a self-report questionnaire (Longo et al., 2008) consisting of eight items: five items refer to ownership (e.g., “It seemed like the rubber hand was my hand”) and three items to location (e.g., “It seemed like my hand was in the location where the rubber hand was”). Participants had to answer on a 7-point Likert scale from −3 (*strongly disagree*) to 3 (*strongly agree*) (see Table 1). We then calculated a mean for each subcomponent (ownership and location), as well as a global score. Higher scores indicate higher illusion. The proprioceptive drift consists of measuring the extent to which the participants perceive their hand and the fake hand as being closer after the stimulation. The drop in skin temperature of the participant’s own hand was measured by subtracting the temperature after the stimulation from the temperature before the stimulation. Lower values indicate a lower increase of temperature and thus a higher illusion.

**Table 1:** **Rubber hand illusion questionnaire statements (−3, strongly disagree to 3, strongly agree)**.

	−3	−2	−1	0	1	2	3
It seemed like I was looking directly at my own hand, rather than at a rubber hand
It seemed like the rubber hand was part of my body
It seemed like the rubber hand was my hand
It seemed like the rubber hand belonged to me
It seemed like the rubber hand began to resemble my real hand
It seemed like the touch I felt was caused by the paintbrush touching the rubber hand
It seemed like the rubber hand was in the location where my hand was
It seemed like my hand was in the location where the rubber hand was

### Procedure

#### General Description

A week before the experimental task, participants had to complete the TAS-20 online (Bagby et al., 1994). When they arrived at the laboratory, participants completed the informed consent form and a questionnaire about their age and sex. After that, they continued with the RHI task. During the task, participants wore a black cloth smock to hide their body from view. They then sat at a table opposite the experimenter with a box (on the table) between them.

**Figure 1:**
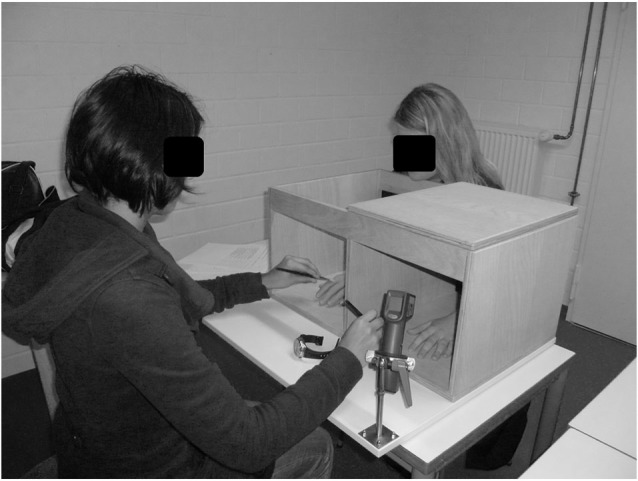
**Experimental design of the rubber hand illusion**.

The dimensions of the box were similar to those described in Tsakiris et al. (2011) (36.5 × 19 × 29 cm [width × height × depth]; see Figure 1). At the front of the left part of the box, a hole was cut, through which the participant placed his or her left hand. The position of the participant’s hand was kept constant by lightly fixing the middle finger to the box with a hook and loop fastener. The top of the left part was closed. The top of the right part of the box was open, through which the participant could see a prosthetic left hand. The entire back side of the box was open to allow the experimenter to brush the participant’s hand and the rubber hand.

#### RHI Induction

After the instruction to place their hand inside the box, participants underwent a pre-induction location judgment. The experimenter placed a removable cover (80 × 29 cm) on top of the box so that the rubber hand could not be seen by the participants. Then, participants were asked to verbally indicate (in cm) the position of the middle finger of their left hand by using a ruler that was placed on the cover of the box. Importantly, the starting position of the ruler was not kept constant from trial to trial in order to prevent participants from repeating responses of prior trials. Subsequently, we measured the temperature of the participant’s left hand using an Infrared Thermometer (Maplin, UK) pointing to the knuckle of the index finger.

After these measures, the cover was raised and the induction phase started with either the synchronous or the asynchronous condition. The order of the conditions was counter-balanced such that half of the participants started with the synchronous condition and the other half with the asynchronous condition. In both conditions, the index fingers of the fake hand and of the participant’s hand were brushed with two identical paintbrushes. The induction phase lasted 120 s and the fingers were brushed with a frequency of approximately 1 stroke per second. In the synchronous condition, the participant’s finger was brushed at the same time than the rubber hand’s finger, whereas in the asynchronous condition, the fingers were brushed alternatively.

After the induction phase (synchronous and asynchronous), the experimenter placed the removable cover on top of the box and measured the post-induction temperature. This was followed by a proprioceptive location judgment. The participant then removed his or her hand from the box and completed the self-report questionnaire. The same procedure was used when testing the illusion in the other condition.

The proprioceptive drift is measured by subtracting the position of the finger reported by the participant before the stimulation from the position of the finger reported by the participant after the stimulation. Higher values indicate a higher drift towards the rubber hand. The temperature drift is measured by subtracting the temperature of the finger before the stimulation from its temperature after the stimulation. Lower values indicate a higher drift towards the rubber hand.

### Data Analysis

Statistical analyses were performed by using the SPSS software package version 18 (SPSS Inc., 2009). Kolmogorov-Smirnov tests showed that all factors were normally distributed (*p*s > 0.11), except for the subjective reports of location after asynchrony stimulation and the temperature after synchrony stimulation (*p*s < 0.05). Therefore, non-parametric analyses (Wilcoxon signed-ranks) were conducted in order to determine the effect of condition (i.e., synchrony vs. asynchrony) on subjective reports of location and on temperature. The other condition comparisons were based on repeated measures. We used Pearson correlations to investigate whether alexithymia is associated with proprioceptive and subjective shifts towards the fake hand. Finally, we used partial correlations to control for the influence of order (synchrony vs. asynchrony) on the association between proprioceptive drift and alexithymia. Data of more than 2.5 standard deviations below or above the participant’s mean in terms of proprioceptive shift and subjective reports were discarded as outliers (seven participants). Forty-three participants (*M*_age_ = 23.10, *SD*_age_ = 4.33; 37 women) were thus included in the analyses.

## Results

### Alexithymia Level

The mean total score on the TAS-20 was 41.50 (*SD* = 9.07, range = 26–68). Participants presented a mean score of 13.42 (*SD* = 3.95) on the DIF, 10.78 (*SD* = 3.55) on the DDF, and 17.29 (*SD* = 4.00) on the EOT.

### RHI Task

Because the order of the condition induction had an effect on proprioceptive drift [for the asynchronous condition, *F*_(1,42)_ = 6.77, *p* = 0.01; asynchronous as first condition: *Mean (SD)* = −1.32 (2.08); asynchronous as second condition: *Mean (SD)* = 0.33 (2.08)] we added order as a covariate in the repeated measure for this measure only.

#### Subjective Feeling of the Illusion (Table 2)

There was a significant main effect of condition, *F*_(1,42)_ = 72.70, *p* < 0.001; *d* = 1.20. Following synchronous stimulation, subjective reports of body illusion were higher than following the asynchronous stimulation. For the two subcomponents of the subjective experience, results indicate a main effect of condition on location *(Z* = −4.97; *p* < 0.001, *d* = 1.19) and ownership (*F*_(1,42)_ = 57.82, *p* < 0.001, *d* = 1.63) such that relative to the asynchrony condition, the synchrony condition led to higher reports that the fake hand and the person’s real hand were closer and that the fake hand was the participants’ own hand.

**Table 2:** **The rubber hand illusion: Mean (Standard Deviation) and correlations with the TAS-20 factors and total score**.

		Proprioceptive shift			Subjective shift (global)			Subjective shift (ownership)			Subjective shift (location)		
		Synchrony	Asynchrony	Shift	Synchrony	Asynchrony	Shift	Synchrony	Asynchrony	Shift	Synchrony	Asynchrony	Shift
Mean		0.01	−0.51	0.52	−0.69	−2.20	1.51	−0.53	−2.04	1.51	−0.95	−2.46	1.51
(SD)		(2.19)	(2.22)	(2.90)	(1.45)	(0.85)	(1.16)	(1.64)	(1.02)	(1.37)	(1.46)	(0.78)	(1.30)
TAS-20	DIF	−0.04	−0.19	0.12	−0.03	0.03	−0.06	−0.01	−0.04	0.02	−0.06	0.16	−0.16
	DDF	0.02	−0.18	0.15	−0.34*	−0.10	−0.36*	−0.31*	−0.14	−0.28	−0.31*	0.02	−0.35*
	EOT	−0.08	−0.12	0.03	−0.42**	−0.05	−0.50****	−0.38*	−0.07	−0.42***	−0.41**	0.00	−0.44***
	TOT	−0.04	−0.21	0.12	−0.33*	−0.05	−0.38*	−0.29	−0.10	−0.29	−0.33*	0.08	−0.40**

Note. TAS-20, Toronto Alexithymia Scale; DIF, difficulty identifying feelings; DDF, difficulty describing feelings; EOT, externally oriented thinking; TOT, TAS-20 total score.****p = 0.001. ***p ≤ 0.005. **p < 0.01. *p < 0.05.

#### Proprioceptive Drift (Table 2)

The main effect of condition was significant, *F*_(1,41)_ = 4.21, *p* = 0.047, *d* = 0.24. Following synchronous stimulation, the proprioceptive drift mean was higher than following the asynchronous stimulation. There was thus a lower drift towards the rubber hand in the asynchronous condition than in the synchronous condition.

#### Temperature

There was no main effect of condition on the temperature drift (*Z* = −1.23; *p* = 0.22). In the synchronous condition, the mean skin temperature drift was 0.09°C (*SD* = 0.45) and 0.24 (*SD* = 0.52) in the asynchronous condition.

### Correlations Between the RHI and the TAS-20 (Table 2; Figure 2)

We calculated correlations between alexithymia factors, proprioceptive and subjective drifts, as well as between alexithymia factors and proprioceptive and subjective shifts (Tsakiris et al., 2011). The proprioceptive shift refers to the increase in proprioceptive drift during the synchronous relative to the asynchronous condition. This shift was calculated by subtracting the proprioceptive drift in the asynchronous condition from the proprioceptive drifts in the synchronous condition. Higher scores refer to a higher drift towards the hand when the visual and tactile simulations are correlated (vs. not correlated). We also calculated the shift in terms of subjective reports by using the same rationale (i.e., subtracting the subjective reports of the illusion in the asynchronous condition from the subjective reports of the illusion in the synchronous condition). Higher scores refer to a stronger impression that the rubber hand was the participant’s hand when the visual and tactile simulations are correlated (vs. not correlated). The temperature shift was not calculated because there was no main effect of condition on temperature. For the proprioceptive shift, we used partial correlations to control for order.

**Figure 2:**
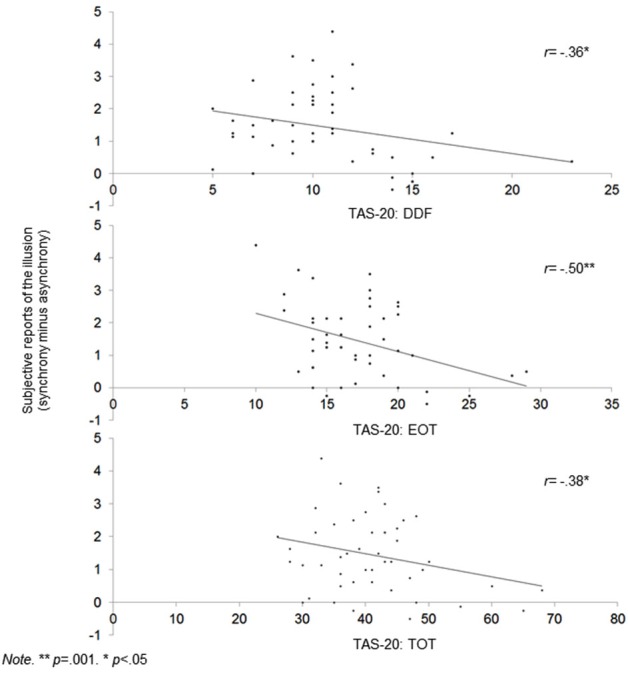
**Scatterplots showing correlations between alexithymia (TAS-20 DIF, EOT and Total score) and global subjective shift**.

Table 2 indicates the following:

1. The DDF, EOT, and TAS-20 total scores were correlated with a lower RHI effect at a subjective level during synchrony condition only. Table 2 also shows that the DDF, EOT, and TAS-20 total scores are correlated with higher reports that the rubber hand was the participants’ hand (ownership) and that their hands and the rubber hand were closer together (location) during synchrony condition. Of importance, even when the significance threshold of these correlations was adjusted for multiple comparisons (Holm-Bonferroni sequential correction), the correlations between EOT and subjective shift remained significant.
2. No alexithymia factor was associated with the proprioceptive shift when controlling for order.

## Discussion

The aim of the study was to test, for the first time, whether alexithymia constitutes a top-down factor that modulates multisensory integration.

### RHI Induction

In line with previous research, we replicated the RHI effect at a subjective level. We showed that relative to the asynchronous condition, the synchronous condition led participants to report a higher illusion that the fake hand was theirs (ownership) and that their hand and the rubber hand were closer together (location). This was confirmed at a behavioral level as we showed that after the synchronous induction period (vs. asynchronous), participants perceived their hand and the rubber hand as being closer together than they really were. Therefore, the present study replicates the original illusion at a subjective as well as at a behavioral level (Botvinick and Cohen, 1998; Tsakiris and Haggard, 2005).

However, contrary to our hypothesis, we did not find an effect of condition in terms of physiological regulation of the body, as previously found in the literature (i.e., temperature; Moseley et al., 2008; Kammers et al., 2011). However, other studies also failed to replicate this effect (i.e., David et al., 2014) and, more importantly, the relevance of this measure as a correlate of subjective experience of the illusion has been questioned (e.g., Rohde et al., 2013).

Our findings thus confirm that multisensory stimulation leads to a greater ownership over a fake hand, at subjective and behavioral levels. Nevertheless, even though the present results support the RHI, the pattern of results does not follow the usual direction. Indeed, the literature shows that synchronous stimulation leads to greater subjective reports of the illusion (above 0) and to a greater drift towards the rubber hand (above 0), and also that asynchronous stimulation leads to lower reports of the illusion (below 0) and to a reduced or no drift towards the rubber hand (e.g., Botvinick and Cohen, 1998; Tsakiris and Haggard, 2005; Rohde et al., 2011; Kalckert and Ehrsson, 2012). Therefore, in contrast to previous findings, our results show a drift away in the asynchronous condition rather than a drift towards the hand in the synchronous condition. These incontinences may be partly accounted for by methodological differences. Indeed, although we used the same instructions and the same procedure as Tsakiris et al. (2011) and carefully selected the prosthetic hand for its medium size and its light color, these two characteristics are known to influence the RHI (Pavani and Zampini, 2007; Farmer et al., 2012). Specifically, these studies show that a darker and smaller rubber hand can reduce the illusion during the RHI. Of importance, the distance between the two hands might have been even more critical. Whereas in our study the distance between the fake and the real hand was 31.50 cm, previous findings (Lloyd, 2007) suggest that a distance of 30 cm might be the threshold upon which the illusion significantly decreases. Our setup might thus have led the participants to not present an ownership illusion over the fake hand during synchrony condition. Future studies should thus replicate our findings by reducing the distance between the fake and real hand to examine its influences on the present results.

### RHI Induction and Alexithymia: Subjective Level

Concerning alexithymia scores, TAS-20 scores are within the normal range in German populations (Müller et al., 2003). In relation to its influence on the RHI, scores at the TAS-20 total, DDF and EOT factors were associated with lower reports of illusion during the synchronous stimulation. Importantly, this effect was due to a decrease of the RHI in the synchronous condition and not due to a relative increase of the RHI in the asynchronous condition. Our findings thus demonstrate that high alexithymia scorers integrate two simultaneous sensory and proprioceptive events into a single experience (i.e., multisensory integration) to a lesser extent than low scorers.

One can postulate that two mechanisms related to body awareness may account for this effect. On the one hand, the lower influence of visual information on the RHI in high alexithymia scorers may support the idea that they preferentially rely on another channel, namely tactile input. Due to hypersensitivity to tactile stimulation (e.g., Lumley et al., 2007), according to which high alexithymia scorers present higher sensitivity to somatic sensations, high alexithymia scorers may more heavily rely on the tactile sensory input than on other inputs. This will subsequently lead them to be less biased by the influence of synchronicity on the proprioceptive perception of their hand, thus resulting in lower RHI. On the other hand, high alexithymia scorers may give more priority to their proprioceptive information over visual and/or tactile inputs. This would have led them to be less influenced by information provided by other modalities. To our knowledge, no research has yet investigated whether alexithymia modulates proprioception or the influence of visual/tactile information on proprioception. Future studies are thus necessary to test these possible underlying mechanisms accounting for the association between alexithymia and lower RHI. For instance, one should test whether alexithymia modulates the size-weight illusion (Charpentier, 1891), which examines the interaction between visual and proprioceptive inputs. Specifically, the latter is based on the expectation that large objects are heavier. Therefore, if alexithymia is associated with greater priority to proprioception, high alexithymia scorers should present lower illusions that large (small) objects are heavier (lighter). If alexithymia is associated with greater priority to visual inputs, high alexithymia scorers should present higher illusions that large (small) objects are heavier (lighter).

The investigation of these research questions would also provide a better understanding of previous RHI findings in clinical and non-clinical populations that are characterized by high levels of alexithymia. Indeed, in clinical populations characterized by high levels of alexithymia, the RHI is either reduced (e.g., ASD; Cascio et al., 2012) or increased (e.g., schizophrenia; Thakkar et al., 2011). This has also been shown in healthy populations in which psychopathological traits are positively associated with alexithymia and with reduced (somatoform symptoms; Miles et al., 2011) or increased RHI (psychosis-proneness; Germine et al., 2013). It seems thus that alexithymia may not constitute the explanatory factor accounting for all these effects. Rather, we argue that one of the two mechanisms previously described (visual or proprioceptive prioritization) may explain these results.

### RHI Induction and Alexithymia: Behavioral Level

Contrary to the results found at a subjective level, no alexithymia factor was associated with a proprioceptive shift. Alexithymia is thus characterized by a decoupling between the subjective and behavioral correlates of the RHI. This is in line with previous studies that highlighted discrepancies between (significant) subjective and (non-significant) behavioral responses in alexithymia. Indeed, although the decoupling pattern is not consistent across studies (i.e., over- or underestimation), many studies revealed a discrepancy between the subjective and physiological responses to emotional or stressful stimuli (e.g., Morrison and Pihl, 1990; Friedlander et al., 1997; Grynberg et al., 2012). At a theoretical level, the present findings support the “decoupling theory” (e.g., Papciak et al., 1985) according to which alexithymia is associated with a dissociation between somatic activity and the subjective reports of this activity. Therefore, even though our sample size prevents us to empirically test this claim by comparing correlations between subjective and behavioral responses separately in high and low alexithymic scorers, the present study extends these findings by confirming disrupted self-awareness in alexithymia.

It is worth mentioning that the absence of correlations between alexithymia and proprioceptive shift could be partly accounted for by an association between EOT and a mislocalization prior to the induction; high EOT scorers indeed initially located their hand farther from the rubber hand than low EOT scorers (*r* = −0.33; *p* = 0.033). Because this mislocalization is part of the measure of proprioceptive shift, it is impossible to control for it. However, this may have biased the proprioceptive shift, potentially explaining the absence of correlations between this measure and alexithymia.

### Implications

At a theoretical level, this study supports the evidence that self-awareness is a multidimensional construct that encompasses several components that are associated one with another (e.g., emotional, interoceptive and exteroceptive). The interdependence of the subdimensions of self-awareness has been previously demonstrated in studies that revealed an association between interoceptive awareness and proneness to show the RHI effect (Tsakiris et al., 2011). Furthermore, the present study supports previous data showing that alexithymia is associated with impaired interoceptive (Herbert et al., 2011) and exteroceptive awareness (De Berardis et al., 2007).

At a clinical level, this study provides support for interventions in high alexithymia scorers that go beyond the improvement of emotional competences (e.g., Levant, 2001). Training alexithymic patients (particularly those with high EOT levels) to pay more attention to signals arising from different locations may improve their multisensory integration and subsequently their body awareness. Eventually, this may reduce the discrepancy between the different components (subjective, physiological or behavioral) of body experiences and thus increase their abilities to regulate the latter.

## Conclusion

This study is the first to show that alexithymia is associated with a distortion of body representations and with a lower integration of multisensory inputs. Future studies are necessary to investigate whether this effect results from higher reliance on visual or on proprioceptive inputs.

## Conflict of Interest Statement

The authors declare that the research was conducted in the absence of any commercial or financial relationships that could be construed as a potential conflict of interest.
